# Chemical Ecosystem Selection on Mineral Surfaces Reveals Long-Term Dynamics Consistent with the Spontaneous Emergence of Mutual Catalysis

**DOI:** 10.3390/life9040080

**Published:** 2019-10-23

**Authors:** Lena Vincent, Michael Berg, Mitchell Krismer, Samuel T. Saghafi, Jacob Cosby, Talia Sankari, Kalin Vetsigian, H. James Cleaves, David A. Baum

**Affiliations:** 1 Wisconsin Institute for Discovery, University of Wisconsin-Madison, Madison, WI 53715, USA; lvincent3@wisc.edu (L.V.); mfberg5@gmail.com (M.B.); mjkrismer@outlook.com (M.K.); ssaghafi@medicine.wisc.edu (S.T.S.); jcosby@wisc.edu (J.C.); tsankari@wisc.edu (T.S.); vetsigian@wisc.edu (K.V.); 2 Department of Bacteriology, University of Wisconsin-Madison, Madison, WI 53706, USA; 3 Geophysical Laboratory, The Carnegie Institution for Science, Washington, DC 20015, USA; henderson.cleaves@gmail.com; 4 Earth-Life Science Institute, Tokyo Institute of Technology, Ookayama, Meguro-ku, Tokyo 152-8550, Japan; 5 Blue Marble Space Institute for Science, Seattle, WA 97154, USA; 6 Institute for Advanced Study, Princeton, NJ 08540, USA; 7 Department of Botany, University of Wisconsin-Madison, Madison, WI 53706, USA

**Keywords:** autocatalysis, chemical ecosystem selection, mineral surfaces, mutual catalysis, prebiotic chemistry, origins of life

## Abstract

How did chemicals first become organized into systems capable of self-propagation and adaptive evolution? One possibility is that the first evolvers were chemical ecosystems localized on mineral surfaces and composed of sets of molecular species that could catalyze each other’s formation. We used a bottom-up experimental framework, chemical ecosystem selection (CES), to evaluate this perspective and search for surface-associated and mutually catalytic chemical systems based on the changes in chemistry that they are expected to induce. Here, we report the results of preliminary CES experiments conducted using a synthetic “prebiotic soup” and pyrite grains, which yielded dynamical patterns that are suggestive of the emergence of mutual catalysis. While more research is needed to better understand the specific patterns observed here and determine whether they are reflective of self-propagation, these results illustrate the potential power of CES to test competing hypotheses for the emergence of protobiological chemical systems.

## 1. Introduction

A critical question in origins of life research is how chemicals first became organized into systems capable of self-propagation and adaptive evolution. While some have proposed that the first evolvers were self-replicating RNA molecules [1], it is also possible that evolution was initiated with the emergence of mutually-catalytic systems (MCSs); sets of chemicals, perhaps including RNA molecules, that could promote each other’s formation such that the MCS self-propagated [2,3,4,5]. In cases where multiple MCSs interacted, for example, by competing for food or cross-feeding, the total ecosystem could evolve by the addition or removal of MCSs as a result of rare reactions or other perturbations. Furthermore, if such evolution could occur, primordial selection would tend to favor ecosystems whose MCSs were better at self-propagating or were able to better withstand environmental fluctuations, or both [2,5]. However, such ecosystem evolution would require that the functional chemicals of MCSs maintained spatial proximity to one another [5]. There are three main ideas for how such proximity could have been maintained initially; enclosure in a spontaneously formed compartment, such as a protocell [3,6,7], adsorption onto a mineral surface [5,8,9], or through non-covalent associations among the cooperating species [10,11]. To evaluate the ecosystem-first perspective and the strengths and weaknesses of these hypothesized spatial structuring mechanisms, experimental strategies are needed to detect the emergence of individual MCSs and evolvable ecosystems of MCSs in the laboratory. Note that we will use the term “mutual catalysis”, which is more or less synonymous with network autocatalysis, as a general term for non-linear chemical systems showing positive feedback, regardless of whether they contain specific catalysts or chemical compounds that directly catalyze their own production.

Several approaches for exploring the emergence of self-propagating entities under laboratory conditions have been proposed, including the application of environmental cycling (in temperature, hydration state, etc.) to force systems out of equilibrium and drive the appearance of life-like chemical entities. One example is wet-dry cycling in which initially aqueous solutions containing dissolved monomers are exposed to cycles of hydration and dehydration to promote polymerization and assembly [12,13,14]. Such experiments are often recursive because the products of early dehydration steps are retained for subsequent cycles. Similarly, recursion has been deployed in prebiotic synthesis experiments to generate increasingly complex chemical assemblages [15,16,17]. The advantage of recursion is that non-linear accumulation of products over cycles can provide evidence for mutual catalysis. However, insofar as simple recursion entails retention of all products from earlier generations, the ability to detect mutually catalytic systems is made more difficult by the presence of a background of chemical species showing simple linear, and hence non-autocatalytic, accumulation.

A potentially more powerful strategy for detecting mutual catalysis is recursion-with-dilution, in which systems are seeded with the products from a previous iteration, but those seeds are diluted with fresh ingredients. Indeed, serial dilution is the standard approach in microbial experimental evolution [18] and has been deployed with *in vitro* genetic systems to select for more effective self-propagators, the classic example being the experimental evolution of RNA sequences in the presence of Qβ-replicase [19]. Such a serial dilution approach has great potential for origins of life research since it could be used to enrich for those mutually catalytic systems whose rate of self-propagation exceeds the rate of dilution [20]. Nonetheless, despite suggestions that this might be worth considering [20,21,22], we are not aware of prior experiments that have deployed recursion-with-dilution to impose artificial selection on prebiotic chemical mixtures to detect the emergence of self-propagating networks of small molecules.

**Figure 1:**
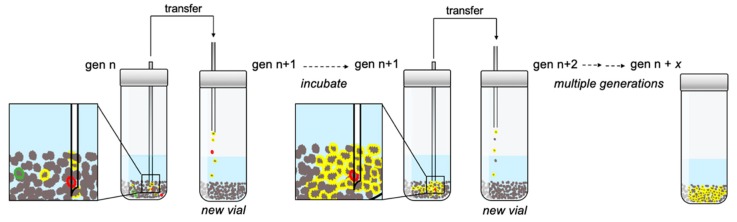
Serial transfer with dilution in a chemical ecosystem selection (CES) experiment has the potential to enrich for mineral-associated mutually catalytic systems, MCSs, that can self-propagate. Each vial contains a synthetic “prebiotic soup” and a population of mineral grains. After an incubation period, a subset of the mineral grains, with or without solution, is transferred to a new vessel containing fresh reagents and virgin grains. The process is repeated over many generations such that mineral-associated MCSs that can self-propagate faster than the rate of serial dilution will become enriched. Furthermore, if multiple MCS variants emerge with different chemistries and colonizing potentials (denoted by different colors), MCSs that are better at colonizing new mineral grains (e.g., the yellow variant in the figure) will tend to dominate over serial transfers.

Chemical ecosystem selection (CES) is a recursion-with-dilution strategy (Figure 1) that can be configured to detect life-like chemical systems based not on the appearance of particular chemical species, but on systematic changes over transfers in emergent chemical proxy traits [20]. Experiments can be conducted under various environmental conditions and can include many different input chemicals, allowing for exploration of diverse hypotheses as to microenvironments that might support the emergence of self-propagating and evolvable ecosystems. However, despite its appeal, it remains to be shown that CES is practical or that it has the potential to detect self-propagating chemical systems. Here, we set out to pilot CES using mineral grains and a simulated prebiotic chemical mixture.

To guide our experiments, we focused on one published model for the emergence of MCSs, namely the surface metabolism model, first published more than 30 years ago by Wächtershäuser (1988). In this model, the first self-propagating systems were MCSs of organic compounds adsorbed onto the surface of iron-sulfur minerals that could use replenishing carbon sources in their environment to regenerate their chemical components. Once seeded by key functional species, MCSs would, in principle, be able to use fluxes of food and energy to generate all of their components, resulting in propagation over mineral surfaces [21,22].

The purest test of Wächtershäuser’s hypothesis would be to use chemical mixtures generated from small molecules (methane, carbon dioxide, water, hydrogen cyanide, etc.) under simulated prebiotic conditions [23,24,25,26] and a set of out-of-equilibrium inorganic ions resembling those that might have been present in or around Hadean hydrothermal vents [27,28,29,30,31]. However, for practical reasons, and as a simple exploratory test, we used a synthetic “prebiotic soup” made by mixing many of the compounds reported in prebiotic synthesis experiments [23,32]. One benefit of making the soup ourselves is that we could enrich it in ways that might increase the chances of a positive result, especially through the addition of potential sources of chemical energy. We reasoned that interesting findings obtained using our enriched prebiotic soup (EPS) could later be evaluated for prebiotic plausibility by seeing if similar dynamics emerge when omitting less plausible chemicals. It is worth noting here that, although the CES framework itself is chemically agnostic and does not require a commitment to any particular chemical process, the deployment of metrics and proxies to track changes and search for evidence of MCSs does require focusing on specific chemical attributes. In the experiments described here, we assayed generic chemical features (pH and UV-Vis absorbance) and orthophosphate concentration, a potential indicator of energy flux derived from ATP, which we included in the EPS.

Here, we describe our CES protocol in sufficient detail that other research groups can conduct similar experiments. We report the results of a series of experiments with EPS and pyrite grains, as these illustrate patterns that are suggestive of the emergence of non-linear chemical systems. These include systematic changes over rounds of transfer (“generations”) and a long-term boom-and-bust pattern that hints at the emergence of a dynamically-maintained, self-propagating chemical ecosystem. While more research is needed to understand the preliminary results reported here, including whether the observed non-linear chemistry indicates the appearance of one or more MCSs capable of self-propagation through surface-associated mutual catalysis, these results illustrate the potential power of CES experiments as a means to detect emergent self-propagating protobiological systems.

## 2. Materials and Methods

### 2.1. Enriched Prebiotic Soup (EPS) Preparation

The composition of the EPS was guided by what one might expect to find if compounds such as those obtained in Miller-Urey-type spark discharge experiments [25] were dissolved in an ocean and concentrated. We recognize that there is considerable debate regarding which compounds are “prebiotically plausible” given the uncertainties about early Earth chemodynamics [23,32], but on first principles, the possibility of one or more MCSs emerging should be increased by including the largest diversity of organic species possible [33] and by providing multiple alternative sources of chemical free energy. To that end, we supplemented the solution with an out-of-redox-equilibrium salt pair (NH_4_^+^ and NO_3_^-^), adenosine triphosphate (ATP), and the strong oxidant ammonium persulfate (APS). The latter has been shown to promote the non-enzymatic production of several metabolically important species [34]. We also included nicotinamide for its possible early role in facilitating prebiotic energy transduction [7], and pantetheine, a possible phosphate-free precursor of cofactor-A [35,36].

The EPS was prepared as two individual solutions (EPS I and EPS II), the compositions of which are summarized in Table 1, to limit reactions between high-energy compounds and the other components as much as possible prior to use in experiments. APS was not included in either stock solution but was added just prior to experiments. A more detailed list of all compounds added to the EPS is provided in the Supplementary Materials (Table S1).

To prepare the solutions, sterile nanopure water was bubbled with N_2_ gas to remove O_2_. All solid and liquid compounds except for APS were added to the de-oxygenated water and allowed to dissolve for one h with magnetic stirring at room temperature (23.5–25 °C). The solutions were then buffered with 1 N NaOH (EPS I to pH 7.5; EPS II to pH 7.8) and filter-sterilized using 0.2 μm polyethersulfone (PES) filter units (VWR International; Radnor, PA; 73520-986). Aliquots of the filtered solutions were dispensed in a biosafety cabinet using sterile 60 mL syringes into pre-autoclaved 100 mL type 1 borosilicate glass vials that were already sealed with crimped aluminum seals over butyl rubber septa. The filled vials were chilled overnight at 4 °C before being stored at −20 °C. Prior to usage, frozen aliquots were thawed at 4 °C overnight and subsequently handled on ice.

### 2.2. Pyrite Powder Preparation

A large number of minerals have been implicated in various aspects of the origin of life [37,38], but pyrite has been of particular interest because (1) it was likely abundant on early Earth in many settings, including hydrothermal vents [8,9,39], (2) it can adsorb many biological building blocks [40,41,42,43], (3) it can catalyze several kinds of biologically important reactions [44,45,46,47], and (4) it is structurally similar to the catalytic cores of many highly conserved biological enzymes [9,48,49,50,51].

Natural pyrite (Ward’s; Rochester, NY, USA; # 470118-152) was mechanically pulverized using a jawcrusher and disc mill fitted with low-phosphorus carbon steel plates (Bico Braun International; Burbank, CA, USA; part number UA-81/82). The resulting powder was size restricted to <150 μm using a stainless steel 100-mesh sieve. We then used a previously published protocol to wash the powder and remove fine dust and oxidized layers [52]. Briefly, the pyrite powder was sonicated for 1 min in a ~1:1 volume ratio with 100% ethanol 10–15 times until the decanted ethanol was clear, and then mixed in a ~1:1 volume ratio with 0.5 M nitric acid (ACS grade; Fisher Scientific; Hampton, NH, USA; A200-500) for 60 s. The acid-washed powder was then rinsed three times with nanopure water and, finally, washed once with 100% ethanol to prevent re-oxidation. Grains were dried, sieved with a 250-mesh sieve to remove particles smaller than 75 μm, and stored in an anaerobic atmosphere (90% N_2_, 10% CO_2_).

The identity of the mineral grains was evaluated by X-ray diffraction on a RIGAKU D/Max Rapid II instrument (50 kV; 50 mA; 60 min exposure). The resulting diffraction patterns (Figure S1A) were identified using Jade software and a Powder Diffraction File from the International Centre for Diffraction Data (ICDD). The grains were also imaged on a FEI Quanta 200 scanning electron microscope in low-vacuum mode (Figure S1B) (30 kV; 5.0 spot-size; 3.0 torr) to assess purity. The extent of pyrite oxidation after acid-washing was determined by suspending a small amount of powder in anaerobic water and measuring the amount of sulfate released by ion chromatography as compared to a standard curve (Figure S2). The acid-washed pyrite powder used in the experiments described here released << 0.05 mM sulfate after equilibrating ~0.2 grams of pyrite in anaerobic nanopure water overnight at room temperature.

### 2.3. Chemical Ecosystem Selection

Our implementation of CES entailed incubating EPS with natural pyrite grains for a defined period, after which a fraction of the grain-soup slurry was transferred to a new vial containing fresh EPS and grains (Figure 1). We reasoned that if an MCS were to nucleate on a mineral grain and propagate from grain to grain faster than the rate of dilution, it would be enriched over generations in the overall reaction volume. Furthermore, in the event that more than one kind of MCS formed, either simultaneously or over time as a result of rare reactions that seeded alternative MCSs, then the more rapidly propagating MCSs would tend to be preferentially enriched (Figure 1). This protocol could also enrich MCSs in solution, although surface-associated systems may be more likely [5].

Pyrite powder (200 mg) was added to 4 mL serum vials, which were then sealed and flushed with N_2_ gas for 5–7 s at the highest flux possible without venting needles being ejected. An EPS master mix was assembled containing equal volumes of EPS components I and II (Table S1). APS, which is highly reactive, was added to the master mix to a final concentration of 0.04 mM, and then 1 mL of the master mix was dispensed into each mineral-containing vial using a pistol-grip syringe (Allflex; Dallas, TX, USA; part number 25MR2) and venting needle. The vials were autoclaved for 45 min on a liquid sterilizing cycle with slow exhaust and incubated at room temperature with gentle rocking for 48 h (Reliable Rocking Shaker 55; 100 rocking motions per min).

To perform serial transfers, new vials were prepared with pyrite and 1 mL of EPS, as described for the initial incubation (generation 0). Then, 100 μL of mineral slurry from the previous generation was transferred into the new vials using sterile insulin syringes (BD Biosciences; Franklin Lake, NJ, USA; #305199) fitted with 18G needles. The vials were autoclaved for 45 min on a liquid sterilizing cycle with slow exhaust and then incubated at room temperature with gentle rocking for the next generation. Although sealed with butyl rubber septa and crimped aluminum seals as a precaution against gas diffusion, vials were kept in an anaerobic atmosphere of 90% N_2_ and 10% CO_2_. The period of incubation almost always followed a 2-day, 2-day, 3-day cycle (i.e., Monday, Wednesday, Friday transfers). This incubation time was arbitrarily selected as there was no theoretical reason to assume an ideal generation time. 

After every six generations in experiment 1, we measured the orthophosphate concentration and UV-Vis absorbance of the samples using the protocols described in Section 2.4 below. In experiment 2, we measured orthophosphate concentrations at the end of each incubation period.

In both experiments, we paired experimental lineages with controls every six generations. Controls were assembled with the same reagents at the same time as experimental lineages and set up one generation prior to use so that they had a single transfer in their history at the time of analysis, as contrasted to the higher number of transfers experienced by experimental lineages. Thus, controls used the same solutions and were subjected to the same environmental conditions as experimental lineages but differed in having one rather than 6, 12, 18, or 24 transfers in their past. The statistical significance of differences between control and experimental samples was evaluated with two-tailed, heteroscedastic Student’s t-tests. The statistical significance of differences among controls in experiment 2 was evaluated using a one-way ANOVA and Tukey-HSD post-hoc test implemented in R [53].

### 2.4. Chemical Analysis

The principle of CES is that instead of looking for specific chemical compounds, we assume that a self-propagating chemical system would result in many measurable changes in the chemistry of the solutions within which it is propagating [54]. We have explored multiple proxy traits, including pH, redox state, and ammonium and nitrate concentration, but the experiments reported here only monitored free orthophosphate concentration and UV-Vis absorbance of the bulk solution.

#### 2.4.1. Inorganic Phosphate Assay

Free orthophosphate concentrations were determined using a phosphomolybdate colorimetric test [55]. To perform the assay, mineral slurries were first harvested and filtered into 96-well plates using 0.20 μm hydrophilic polypropylene (GHP) filter plates (VWR International; Radnor, PA, USA; #97052-096) and a vacuum manifold to separate the grains from the bulk solution. 15 μL of the phosphomolybdate reagent (Millipore Sigma; Burlington, MA, USA; MAK030) was added to 100 μL of filtrate, and the reaction was incubated for 30 min at room temperature in the dark. Absorbance at 650 nm was measured using a plate spectrophotometer (BioTek Instruments Synergy HT) and converted to orthophosphate concentration using a standard curve.

#### 2.4.2. UV-Visible Spectroscopy (UV-Vis)

Sample filtrates prepared as described in the previous Section were transferred into UV-transparent 96-well plates (Corning; Corning, NY, USA; part number 07200623) and absorption spectra from 200 to 400 or 700 nm (at 5 nm intervals) were obtained on a plate spectrophotometer (BioTek Instruments Synergy HT). 

### 2.5. Environmental Scanning Electron Microscopy (ESEM)

Mineral slurries were harvested and dried on a vacuum manifold at -200 mbar overnight. The dried powder was then mounted on aluminum pegs using double-sided carbon tape and loaded onto a FEI Quanta 200 in ESEM mode (20–30 kV; 5.0 spot-size; 3.0 torr). To estimate the frequency of microscopic structures visible on pyrite grains, 5–10 random fields at 500× magnification were captured for three experimental and three control replicates and individual pyrite grains were scored for the presence of structures as compared to the total number of grains per field. Figure S5 includes a set images for one control and one experimental replicate to illustrate the scoring method used to estimate the percentage of pyrite grains with visible microscopic structures.

### 2.6. ESEM Experiment with EPS Variants

To ascertain which components of the EPS were required for the formation of microscopic structures on pyrite grains, we incubated natural pyrite with variant EPS soups (no salt, no organics, no ATP, KH_2_PO_4_ instead of ATP, and MgSO_4_ instead of ATP) in a mixture containing five times more EPS than the CES experiments, relative to pyrite grains. Four hundred milligrams of pyrite powder was added to 10 mL serum vials that were sealed using butyl rubber septa and crimped aluminum caps. The vials were flushed with N_2_ gas for 10 s. Equal volumes of modified EPS I and EPS II were mixed, APS was added to a final concentration of 0.04 mM, and 10 mL of this master mix was added into each vial. The vials were autoclaved for 45 min on a liquid sterilizing cycle and incubated at room temperature with gentle rocking under a 90% N_2_, 10% CO_2_ atmosphere. Grains were harvested 2 days after autoclaving for observation under ESEM to evaluate the proportion of pyrite grains with microscopic structures.

### 2.7. Time Course Experiment

To determine whether intergenerational dynamics arise from non-linear chemical reactions within vials in the absence of transfer, we generated time series data for orthophosphate concentration, UV-Vis absorbance, and the proportion of pyrite grains with visible microscopic structures. Given the possibility that any extraction from a vial would change its chemistry, we generated enough vials such that each could be incubated in parallel, with ten sampled destructively at each time point.

Vials containing EPS and pyrite grains were prepared as described in Section 2.3. The vials were autoclaved for 45 min on a liquid sterilizing cycle and incubated in the dark in a thermostatically controlled orbital shaker at 25 ˚C and 100 rpm. Two identical sets were prepared 12 h apart. A subset of randomly selected vials (20 vials at 12-h intervals, 10 vials at all other times) were sampled destructively every 4 h over a 4.5-day period and analyzed using the phosphomolybdate assay and by UV-Vis spectroscopy. Grains were harvested from selected time points and dried on a vacuum manifold for 24 h before being imaged by ESEM and scored for the presence of fractal structures using the same method described in Section 2.6.

## 3. Results

**Figure 2:**
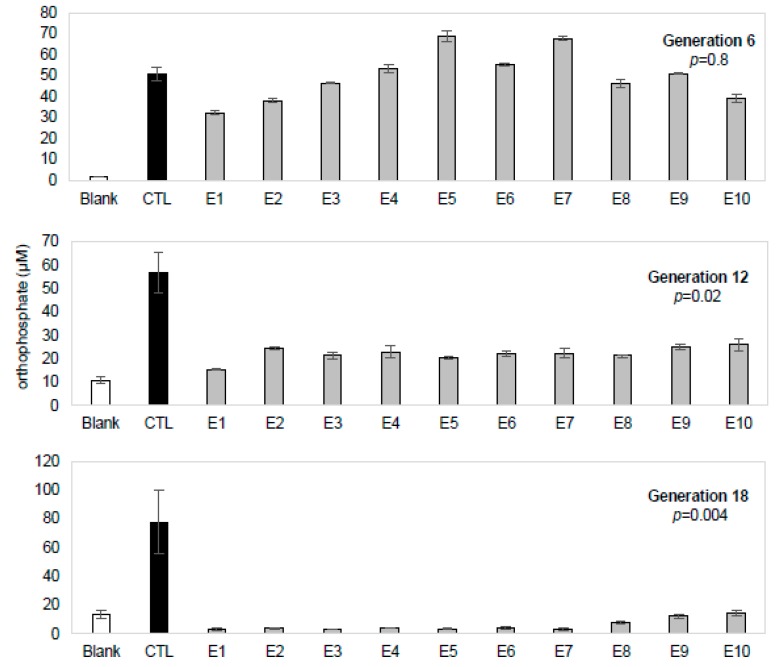
Free orthophosphate concentrations measured in the bulk solution of 10 independent lineages (E1–E10) at the end of generations 6, 12, and 18 compared to a control set (CTL) of 10 replicates that had a single transfer in their history, and starting EPS solution not exposed to mineral (Blank). CTL error bars represent the standard error of the mean for the 10 replicates. E1–E10 error bars correspond to the standard deviation for three measurements made on each experimental lineage. The statistical significance of differences between control and experimental samples was determined using two-tailed, heteroscedastic Student’s t-tests.

One milliliter of a synthetic enriched prebiotic soup (EPS) and 200 mg of pyrite grains were combined in sealed serum vials, with an aliquot of soup-grain slurry transferred to a new vial already containing fresh EPS and grains every 2–3 days. Because 100 µL was transferred at each generation from vials that contained ~1.1 mL of solution (1 mL of fresh EPS plus 100 µL added from the previous generation), this amounts to a transfer of ~9% of the total volume. In an initial experiment, ten lineages were propagated in parallel and, every 6 generations, the amount of free orthophosphate (ΣPO4^3−^ and protonation states) present in vials at the end of a generation was measured in the bulk solution. We tracked orthophosphate because it can be used as a proxy for ATP hydrolysis, a potential source of chemical energy and the sole source of phosphate added to the EPS. To ensure that any changes detected were the result of an extended history of serial transfer rather than variation in the environment or aging of solutions, controls were set up in the same manner as the experimental lineages but allowed only one transfer before being analyzed (with the same reagents and at the same time as the experimental vials). We observed a consistent decline in the amount of orthophosphate present after the incubation period in the ten independent lineages at generations 6, 12, and, 18, while controls showed no such decline (Figure 2). The significant decrease in the orthophosphate concentration relative to controls seen in generations 12 and 18 suggests that a system arises after multiple transfers that renders orthophosphate undetectable by this assay, whether through precipitation, pyrite adsorption, or conversion into inorganic secondary anions or phosphorylated organic compounds.

**Figure 3:**
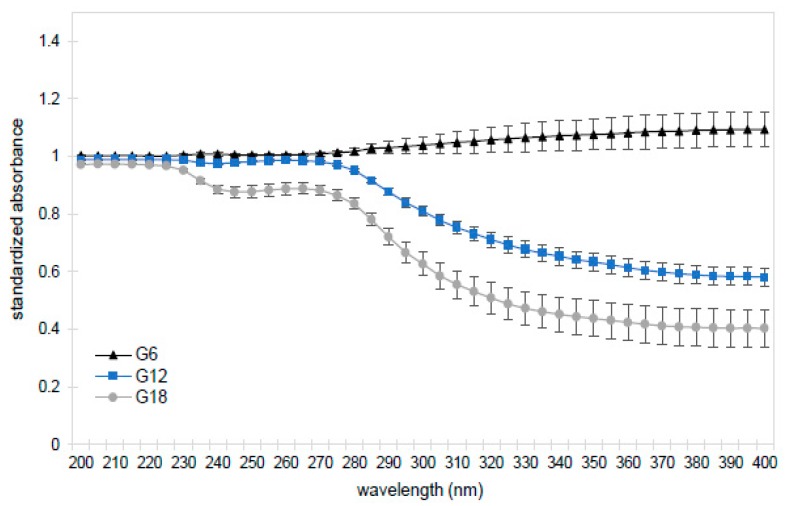
The relative UV-Vis absorbance at generations 6, 12, and 18 of experimental lineages compared to the average absorbance of the corresponding control set (10 identical samples that had only a single transfer in their past). This result suggests that the light-absorbing compounds (including organics) remaining in solution at the end of an incubation period become depleted after multiple serial transfers. (The raw data used to generate this figure can be found in Table S3 and Figure S3).

As many organic compounds in the EPS absorb in the UV-Vis range, including amino acids and nucleobases, absorption spectra for experimental and control lineages were compared to see if any significant changes resulted from the serial transfer protocol. Indeed, we found that the UV-Vis absorbance of the bulk solution in all experimental lineages exposed to 12 rounds of transfers was lower than in the controls at nearly every wavelength measured between 200 and 400 nm (Figure 3, Figure S3 and Table S3). The total UV-Vis absorbance continued to decrease with additional transfers, with generation 18 being just 88% that of controls (1.46 ± 0.10 AU vs. 1.66 ± 0.20; *t*-test, *p*-value <0.05). One interpretation of these data is that, like orthophosphate, light-absorbing organics become depleted from the solution in later generations. We have confidence that these results are not due to microbial contamination as we used pre-sterilized materials, autoclaved the vials at the start of each incubation period, observed no increase in turbidity or anomalies in the absorbance at 600 nm (at which microbial cells typically scatter light), and observed no bacterial cells upon ESEM examination of pyrite grains.

**Figure 4:**
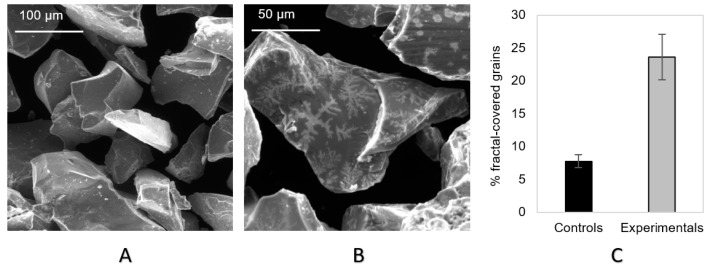
Environmental Scanning Electron Microscopy (ESEM) images of pyrite grains (**A**) from a control sample exposed to a single transfer, and (**B**) from a lineage exposed to 18 transfers. The mean percentage of pyrite grains with visible fractal structures in control and experimental lineages (**C**) was determined from five random fields at 500x magnification. Error bars represent the standard deviation of three replicates. An example set of images used to score grains with visible fractals can be found in Figure S5.

The coincident reduction in the concentration of dissolved orthophosphate and organic compounds in the solution led us to inspect the pyrite grains by ESEM to determine whether precipitates were present. Grains exposed to many rounds of serial transfer had a significant number of visible microscopic structures with a distinctive fractal morphology on their surface, which were rare in control samples (Figure 4), (*t*-test, *p*-value < 0.0001). 

To better understand the dependence of fractal structures on different compounds in the EPS, we incubated pyrite grains with modified soups and counted the proportion of grains with fractals visible after 2 days of incubation (Table 2). We used a higher soup-to-grain ratio than in the transfer experiments to avoid the risk of fractals being present only transiently. We did not observe any visible fractal structures on pyrite grains incubated in versions of the EPS that lacked either the organic or salt fraction, which shows that both are needed for fractal production. Although ATP also appears to be needed, the phosphodiester bonds themselves may not be required since replacement with either potassium phosphate or magnesium sulfate salts at the same molarity as ATP yielded a superabundance of fractal structures. Note that replacing ATP with the same final concentration of either KH_2_PO_4_ or MgSO_4_ (0.32 mM) yielded an EPS variant with a different overall ionic strength and charge density.

To evaluate whether the fractal structures are present on submerged grains or appear only upon drying, pyrite grains were imaged by ESEM while raising and then lowering the water vapor pressure inside the microscope chamber. Fractals were observed to dissolve at high water vapor pressures (between 6.5 and 8.0 torr) and then reappear below 3.5 torr, but with a somewhat altered morphology (Figure S3). This result suggests that fractals form when the salt-rich solution crystallizes in the ESEM chamber at low water vapor pressures and do not exist when the pyrite grains are in solution. Nonetheless, we propose that the fractals indicate the presence of an organic layer on the pyrite surfaces. This is supported by the fact that both organics and salts are necessary for fractal formation, and that NaCl is known to yield similar halite crystal morphologies in the presence of organics [56]. Thus, the fractal crystals are likely generated during drying, but it is possible that organic layers deposited from the EPS soup create a unique environment for their formation. Therefore, the fact that fractals are significantly more abundant in experimental than control samples at generation 18 (*t*-test, *p*-value < 0.001) suggests that pyrite grains in experimental lineages accumulated more organic material on the surface of pyrite grains during an incubation, potentially explaining the observed reduction in UV-Vis absorbance. Additional experiments are needed to test our hypothesis that an organic layer accumulates on the pyrite grains subjected to multiple rounds of transfer and that its presence determines whether or not fractal structures appear upon drying.

**Figure 5:**
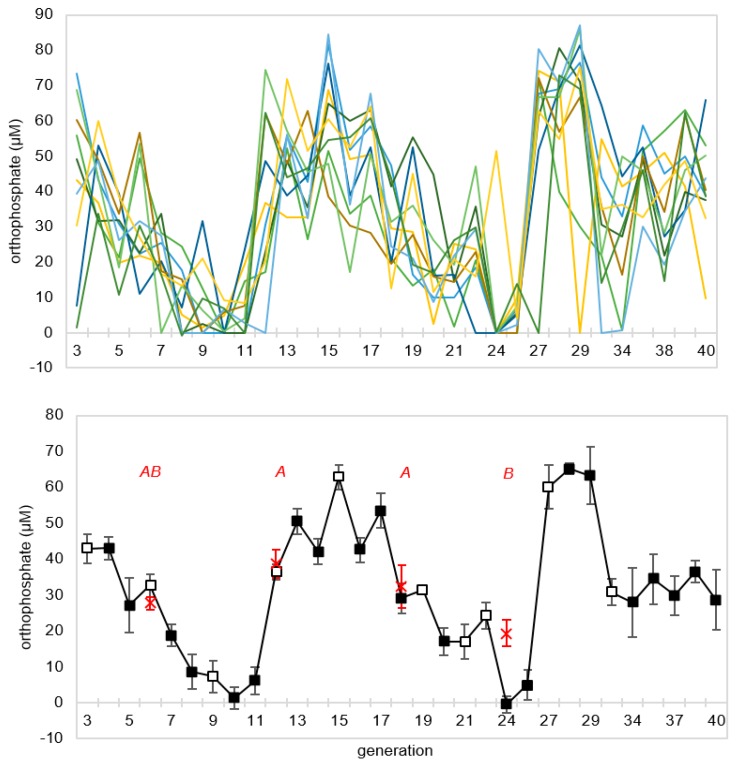
Free orthophosphate concentrations measured in the bulk solution of 10 experimental lineages over 40 generations (**upper panel**). The **lower panel** shows the average of the experimental lineages (error bars = standard error of the mean). The **red crosses** at generation 6, 12, 18, and 24 depict the mean and standard error of 10 controls. The **capital letters** above controls are the output of a Tukey-HSD post-hoc test (different letters signify statistical significance; Table S2). (■) points followed 2-day incubations, and (**☐**) points followed 3-day incubations.

To assess the repeatability of the decline in orthophosphate concentration over generations and to monitor its behavior over more serial transfers, a second CES experiment was set up using the same conditions as the prior experiment, but carried out for 40 generations, with free orthophosphate concentration measured at the end of every generation. As in the first experiment, there was an initial period of declining phosphate, but this decline reversed in all lineages around the tenth generation, only to decline again thereafter (Figure 5). There appeared to be an oscillatory dynamic with orthophosphate concentration minima occurring in generations ~10 and ~24. There was marked generation-to-generation variability within lineages, but the 10 lineages remained reasonably synchronized until approximately generation 30.

**Figure 6:**
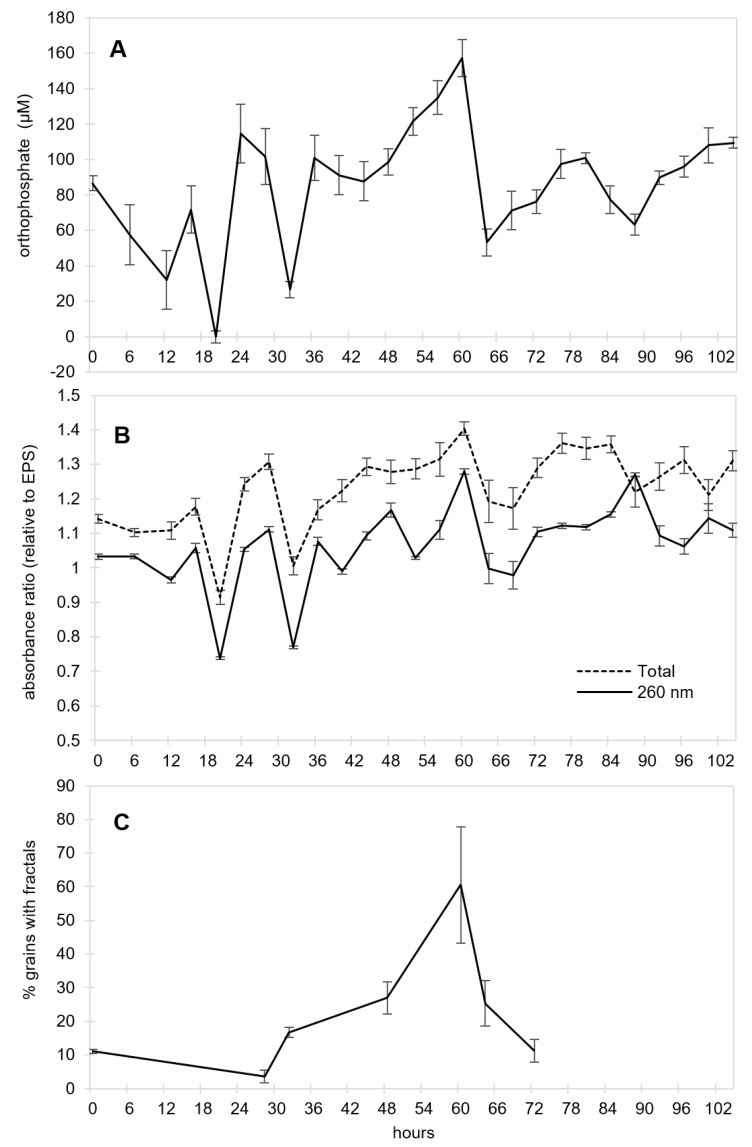
Time course experiment in which 10 replicate vials containing EPS and pyrite grains were sampled destructively every 4 hand assayed for (**A**) orthophosphate concentration, (**B**) total UV-Vis absorbance between 200 and 700 nm relative to the starting EPS soup, and (**C**) mean percentage of grains with visible fractal structures over the first 72 h. Error bars represent the standard error of the mean in panels **A** and **B** and standard deviation of three replicates in panel **C**. Panel **B** also shows the absorbance at 260 nm, which, along with nearby wavelengths in the UV-range, shows more marked oscillations and generally lower absorbance relative to the starting solution.

The dynamic pattern apparent in the long-term CES experiment prompted us to investigate the potential for the EPS/pyrite mixture to display non-linear behavior in the absence of transfers. To do this, we sampled vials destructively every 4 h for 4.5 days to measure the orthophosphate concentration, UV-Vis absorbance (relative to the starting solution), and the percentage of grains with visible fractal structures. We found non-linear changes in all measured proxy traits (Figure 6). Most notably, both the orthophosphate and UV-Vis data revealed oscillations, which were pronounced over the first 36 h. Non-linear changes were sustained far beyond the typical incubation periods used in our CES experiments (48 and 72 h) (Figure 6A,B). These patterns suggest that one or more chemical oscillators spontaneously arose in the EPS/pyrite mixture independently of transfers. Similar to the first transfer experiment (Figure 3), there was a positive correlation between orthophosphate and UV-Vis absorbance, which both had local minima at 20, 32, and 64–68 h. We did not see such oscillations in similar time course experiments carried out in the absence of pyrite (data not shown), although these data may be confounded by the very high orthophosphate concentrations measured in the absence of pyrite.

We observed a gradual increase in the percentage of pyrite grains with visible fractals (Figure 6C), which peaked at around 60 h (midway between a 2-day and 3-day incubation period) after which it declined. These results suggest that surface-associated organics, which are likely needed for fractal structure formation upon drying, build-up in the course of an incubation period, but are unstable if the period of incubation extends too long. The highly controlled nature of this experiment with regards to temperature, light, and shaking regime suggests that the non-monotonic patterns in all three proxy traits, and especially the oscillations in orthophosphate and UV-Vis absorbance, reflect the emergence of non-linear reaction systems that include some positive feedback elements [57,58].

## 4. Discussion

### 4.1. Preliminary Evidence That Mutually Catalytic Systems Can Emerge Through CES with Pyrite

The observed reduction in the concentration of orthophosphate in the first experiment, confirmed by comparing experimental and control lineages, is consistent with our hypothesis that a non-linear chemical system, perhaps an MCS, arose under these conditions. The use of a long autoclaving cycle at each generation combined with the lack of any turbidity, increase in optical density at 600 nm, or visual evidence of microbes under ESEM suggests that these results are not artifacts of microbial contamination. Furthermore, the fact that the loss of free orthophosphate correlates with both the reduction of UV-Vis absorbance and the appearance of fractal structures on mineral surfaces suggests that the onset of this non-linear system is likely to involve surface-associated organics. Thus, while much more work is needed, these data might document the emergence a surface-limited MCS such as that envisaged by in Wächtershäuser’s surface metabolism hypothesis [8,9,59].

The significant difference between experimental and control lineages seen in experiment 1 and at generation 24 in experiment 2 shows that a history of serial transfers, rather than simply environmental variability, alters the chemistry occurring during incubation. Recall that experimental and control vials in these generations utilized exactly the same reagents and were incubated together in the same environment. However, it is important to acknowledge that the unfortunate timing of controls in experiment 2 means that we cannot completely rule out the possibility that the changes observed over generations are due to external factors, such as periodic variations in temperature, light, or aging of the EPS over time. Despite this, because the experimental lineages oscillate between values never reached by any control lineage, we are confident that at least some of the dynamical behavior apparent in the long-term CES experiment is the result of changes induced by serial transfer. However, to improve this protocol in the future, controls could be implemented more frequently (e.g., every three generations) and additional control types, such as lineages propagated without mineral grains, should be included.

If we interpret the decline in residual orthophosphate over the first few generations as an increase in the abundance of an MCS, the latter reversion to high phosphate seen in experiment 2 could reflect the putative MCS’s concentration at the start of an incubation being sufficient that key resources are depleted within the incubation period, resulting in dissolution of the system. Or put another way, the cycling could reflect ecological booms followed by busts, when a putative MCS overshoots its “carrying capacity.” In this regard, it is worth noting that both bust phases were preceded by 3-day incubation periods. It is also interesting that many (7/10) lineages do not undergo a third episode in which the orthophosphate concentration goes to zero. This escape from the oscillatory pattern might be indicative of a transition of some lineages to a new dynamical regime.

Much more information is needed before the ecological boom-and-bust interpretation can be robustly interpreted. Furthermore, these results need to be replicated by other groups, and detailed chemical analyses (e.g., using chromatography-mass spectrometry methods) are required to confirm that the dynamical pattern reflects non-linear organic chemical reaction systems rather than, for example, crystallization. However, supposing that the phenomena we describe were replicated and we could confirm the discovery of a spontaneously forming MCS, this CES protocol is well suited to further investigation. For example, the EPS soup could be modified to clarify which input species are needed to drive the observed dynamical patterns. Among such modifications, the reactants could be simplified to include only chemicals that the community agrees were plausibly present on the prebiotic Earth. Furthermore, examination of the propagation rate at different time points, perhaps combined with experimental manipulation of the environment, could be used to detect long-term changes in propagation rates suggestive of adaptive evolution.

Even if future work rules out there being any MCSs present, or if they are shown to be present but not evolvable, the oscillatory patterns seen in the time course experiment could contribute to ongoing discussions of prebiotic oscillators at the origins of life [57,58,60,61]. In addition to further chemical analyses, future experiments may be aimed at determining whether the intragenerational dynamics apparent in our time course data change when seeded with samples from different generations of a CES experiment or incubated for different periods of time prior to transfer, or both.

### 4.2. CES Can Potentially Enrich Life-Like Chemical Dynamical Patterns in Simulated Prebiotic Conditions

The simplicity of this CES protocol and its potential ability to detect self-propagating chemical systems without prior commitment to a particular chemical process might lead one to view it as highly likely that MCSs can arise and be detected, whether they are surface-associated or in solution. Countering this perspective, our protocol should be sensitive to the rate of propagation of the systems that arise. A system whose rate of propagation is below the rate of serial dilution, which in this case means a rate of proliferation less than about eleven-fold (100 µL in ~1.1 mL) every 2–3 days, would eventually be diluted out of existence by the serial transfer protocol. Conversely, a system that propagated to the maximum degree possible (given the available resources and surface area for colonization) within a single incubation period would not show a trend of change over generations in any proxy trait. Thus, the fact that we observed changes over generations that were similar in 10 replicate lineages, while being distinct from controls, is rather remarkable. One possibility is that we were just lucky with our choice of dilution rate and incubation time. This interpretation is supported by the different timing of the two experiments; the initial phosphate/absorbance decline took ~18 generations in the first experiment, but ~10 in the second. In each case, the declines occurred similarly across replicate lineages, suggesting that the difference was not simply experimental noise. Rather, we suspect that subtle differences between batches of EPS soup, as would arise as stocks age and through measurement errors during soup preparation, could have altered the reactivity of the solutions sufficiently to result in a two-fold rate difference. If so, we can conclude that we were fortunate indeed that the systems emerging had self-propagation rates within the detectable range. Alternatively, given a sufficiently diverse soup, there might exist many potential MCSs such that with almost any transfer/dilution rate at least one MCS could become enriched.

Whether through good luck or unexpected robustness in the protocol, our results suggest that we can enrich for non-linear chemical systems and potentially MCSs using this CES protocol. While a monotonic change over generations is possible without mutual catalysis, for example, if the product of a slow linear reaction became enriched over transfers, the longer-term boom-and-bust pattern detected in experiment 2 is difficult to reconcile with a linear chemical regime. The complex dynamics observed even in the absence of serial transfers indicates extensive non-linearity in our experimental system. Consequently, even without a detailed chemical understanding of the patterns observed here, this study suggests that CES can be used to examine the origins of non-linear chemical systems with the potential for self-propagation.

### 4.3. CES for Systematic Exploration of Competing Origin of Life Scenarios

Whether it is ultimately shown that a self-propagating chemical system has emerged in our experiments using EPS and pyrite or not, there are reasons for deploying CES in many other areas of chemical parameter space. If we have discovered a chemical system capable of self-propagation, and perhaps evolution, then exploration of many other soup-mineral combinations would help clarify the frequency of MCS emergence and, if many conditions do yield MCSs, whether each mixture generates a unique MCS or if multiple starting conditions converge on a small number of robust MCSs. If, instead, our hypothesis of self-propagation is refuted and other processes are found to explain the observed patterns, then there would still be a good reason to use the approach to broadly explore chemical parameter space in the hopes of finding one that *does* demonstrate life-like properties.

Even if these studies are restricted to chemical contexts that could be plausibly present somewhere in primitive planetary environments, there are a huge number of combinations of chemical soups, mineral grains, and environmental variables (e.g., temperature, pressure, light) that could be considered. This search space would become even larger if we aimed to explore chemical mixtures that were modelled upon other real or imagined worlds. Fortunately, the methods needed to implement CES are relatively simple, meaning that they can, in principle, be conducted by research groups lacking sophisticated instrumentation, allowing a broad, community-wide effort to be mounted. The protocol requires standard laboratory equipment (autoclave, spectrophotometer, nitrogen source, glove box, scales, pipettors, vacuum manifold), and the necessary supplies for each step of the protocol are reasonably inexpensive. Additionally, there is the possibility of rendering CES higher throughout, for example by using robotics to automate serial transfer or by developing continuous flow systems. We hope, therefore, that the presentation of this protocol and preliminary results will encourage other scientists to begin such a broad search of chemical parameter space using CES to better understand when and how life-like chemical systems emerge.

## Figures and Tables

**Table 1:** Bulk composition of enriched prebiotic soup (EPS) stocks. See Supplemental Table S1 for details. Equal volumes of soups I and II were mixed just prior to setting up experiments, thereby halving all the concentrations.

**EPS I**		
**Class**	**Compound class**	**Concentration**
Amino acids	All 20 biological amino acids, 2-aminobutyric acid, sarcosine, β-alanine	5.12 mM
Organic acids	Acetic, butyric, lactic, formic, propionic, pyruvic, hydroxybutyric, iminodiacetic, glycolic	2.08 mM
Transition metals	Co(II), Ni(II), Mo(VI), Zn(II), Cu(II)	>1 μM
Inorganic salts	NaNO3, NaHSO3, NH4Cl, NaCl, KCl, MgCl2	~1 M
Other	Glycerol, ethanolamine, methylurea, urea	1.6 mM
**EPS II**		
**Class**	**Compound class**	**Concentration**
Nucleobases *	Adenine, cytosine, thymine, uracil	2.24 mM
Sugars	DL-arabinose, D-ribose, D-xylose, D-glucose	1.6 mM
Cofactors	Pantetheine, nicotinamide	0.48 mM
High-energy compounds	ATP, ammonium persulfate	0.36 mM

* Guanine was not included because of its insolubility in water at near-neutral pH.

**Table 2:** Range of proportions of pyrite grains with fractals incubated with modified EPS soups.

Condition	Fractals
EPS	[14.7%–18.0%]
Minus organics	0%
Minus salts *	0%
Minus ATP	0%
KH_2_PO_4_ instead of ATP	[30.8%–67.1%]
MgSO_4_ instead of ATP	[28.5%–50.0%]

* inorganic sea salts, NaNO_3_, and NH_4_Cl.

## Supplementary Materials

The following are available online at https://www.mdpi.com/2075-1729/9/4/80/s1, Table S1: detailed composition of the EPS soup, Table S2: one-way ANOVA and Tukey-HSD test outputs, Table S3: raw UV-Vis absorbance data for control and experimental lineages at generations 6, 12, and 18, Figure S1: XRD and ESEM analysis of acid-washed pyrite grains, Figure S2: sulfate ion chromatography standard, Figure S3: UV-Vis absorption spectra for control and experimental lineages at generations 6, 12, and 18, and Figure S4: fractal dissolution and recrystallization ESEM images, Figure S5: example of ESEM image scoring strategy as used to estimate the percentage of grains with fractal structures in one experimental and one control replicate.

## References

[1] Gilbert W. (1986). Origin of life: The RNA world. Nature.

[2] Hunding A., Kepes F., Lancet D., Minsky A., Norris V., Raine D., Sriram K., Root-Bernstein R. (2006). Compositional complementarity and prebiotic ecology in the origin of life. Bioessays.

[3] Vasas V., Fernando C., Santos M., Kauffman S., Szathmáry E. (2012). Evolution before genes. Biol. Direct.

[4] Hordijk W., Steel M., Kauffman S. (2012). The structure of autocatalytic sets: Evolvability, enablement, and emergence. Acta Biotheor..

[5] Baum D.A. (2018). The origin and early evolution of life in chemical composition space. J. Theor. Biol..

[6] Morowitz H.J., Heinz B., Deamer D.W. (1988). The chemical logic of a minimum protocell. Orig. Life Evol. Biosph..

[7] Luisi P.L., Walde P., Oberholzer T. (1999). Lipid vesicles as possible intermediates in the origin of life. Curr. Opin. Colloid Interface Sci..

[8] Wächtershäuser G. (1990). Evolution of the first metabolic cycles. Proc. Natl. Acad. Sci. USA.

[9] Wächtershäuser G. (1988). Before enzymes and templates: Theory of surface metabolism. Microbiol. Rev..

[10] Segrè E., Lancet D. (2000). Composing life. EMBO Rep..

[11] Shenhav B., Segrè D., Lancet D. (2003). Mesobiotic emergence: Molecular and ensemble complexity in early evolution. Adv. Complex Syst..

[12] Sibilska I.K., Chen B., Li L., Yin J. (2017). Effects of trimetaphosphate on abiotic formation and hydrolysis of peptides. Life.

[13] Forsythe J.G., Yu S.S., Mamajanov I., Grover M.A., Krishnamurthy R., Fernandez F.M., Hud N.V. (2015). Ester-mediated amide bond formation driven by wet-dry cycles: A possible path to polypeptides on the prebiotic Earth. Angew. Chem. Int. Ed..

[14] Damer B., Deamer D. (2015). Coupled phases and combinatorial selection in fluctuating hydrothermal pools: A scenario to guide experimental approaches to the origin of cellular life. Life.

[15] Colón-Santos S., Cooper G.J.T., Cronin L. (2019). Taming the combinatorial explosion of the formose reaction via recursion within mineral environments. ChemSystemsChem.

[16] Doran D., Abul-Haida Y.M., Cronin L. (2019). Emergence of function and selection from recursively programmed polymerisation reactions in mineral environments. Angew. Chem. Int. Ed..

[17] Mayer C., Schreiber U., Dávila M.J., Schmitz O.J., Bronja A., Meyer M., Klein J., Meckelmann S.W. (2018). Molecular evolution in a peptide-vesicle system. Life.

[18] Lenski R.E., Rose M.R., Simpson S.C., Tadler S.C. (1991). Long-term experimental evolution in Escherichia coli. I. Adaptation and divergence during 2000 generations. Am. Nat..

[19] Mills D.R., Peterson R.L., Spiegelman S. (1967). An extracellular Darwinian experiment with a self-duplicating nucleic acid molecule. Proc. Natl. Acad. Sci. USA.

[20] Baum D.A., Vetsigian K. (2016). An experimental framework for generating evolvable chemical systems in the laboratory. Orig. Life Evol. Biosph..

[21] Baum D.A. (2015). Selection and the origin of cells. BioScience.

[22] Shapiro R. (2000). A replicator was not involved in the origin of life. IUBMB Life.

[23] McCollom T.M. (2013). Miller-Urey and beyond: What have we learned about prebiotic organic synthesis reactions in the past 60 years?. Annu. Rev. Earth Planet. Sci..

[24] Miller S.L. (1953). A production of amino acids under possible primitive Earth conditions. Science.

[25] Miller S.L., Urey H.C. (1959). Organic compound synthesis on the primitive earth. Science.

[26] Keosian J. (1968). The Origin of Life.

[27] Martin W., Russell M.J. (2007). On the origin of biochemistry at an alkaline hydrothermal vent. Philos. Trans. R. Soc. B Biol. Sci..

[28] Russell M.J., Daniel R.M., Hall A.J., Sherringham J.A. (1994). A hydrothermally precipitated catalytic iron sulphide membrane as a first step toward life. J. Mol. Evol..

[29] Russell M.J., Hall A.J. (1997). The emergence of life from iron monosulphide bubbles at a submarine hydrothermal redox and pH front. J. Geol. Soc..

[30] Martin W., Baross J., Kelley D., Russell M.J. (2008). Hydrothermal vents and the origin of life. Nat. Rev. Microbiol..

[31] Von Damm K.L. (1990). Seafloor hydrothermal activity: Black smoker chemistry and chimneys. Annu. Rev. Earth Planet. Sci..

[32] Miller S.L., Cleaves H.J. (2006). Prebiotic chemistry on the primitive Earth. Systems Biology Volume 1: Genomics.

[33] Mossel E., Steel M. (2005). Random biochemical networks: The probability of self-sustaining autocatalysis. J. Theor. Biol..

[34] Keller M.A., Kampjut D., Harrison S.A., Ralser M. (2017). Sulfate radicals enable a non-enzymatic Krebs cycle precursor. Nat. Ecol. Evol..

[35] Goldford J.E., Hartman H., Smith T.F., Segre D. (2017). Remnants of an ancient metabolism without phosphate. Cell.

[36] Keefe A.D., Newton G.L., Miller S.L. (1995). A possible prebiotic synthesis of pantetheine, a precursor to coenzyme A. Nature.

[37] Hazen R.M., Sverjensky D.A. (2010). Mineral surfaces, geochemical complexities, and the origins of life. Cold Spring Harb. Perspect. Biol..

[38] Cleaves H.J., Scott A.M., Hill F.C., Leszczynski J., Sahai N., Hazen R. (2012). Mineral–organic interfacial processes: Potential roles in the origins of life. Chem. Soc. Rev..

[39] Sojo V., Herschy B., Whicher A., Camprubi E., Lane N. (2016). The origin of life in alkaline hydrothermal vents. Astrobiology.

[40] Hatton B., Rickard D. (2008). Nucleic acids bind to nanoparticulate iron (II) monosulphide in aqueous solutions. Orig. Life Evol. Biosph..

[41] Bebié J., Schoonen M.A.A. (2000). Pyrite surface interaction with selected organicaqueous species under anoxic conditions. Geochem. Trans..

[42] Sanchez-Arenillas M., Mateo-Marti E. (2016). Pyrite surface environment drives molecular adsorption: Cystine on pyrite(100) investigated by X-ray photoemission spectroscopy and low energy electron diffraction. Phys. Chem. Chem. Phys..

[43] Pontes-Buarques M., Tessis A.C., Bonapace J.A.P., Monte M.B.M., Cortes-Lopez G., De Souza-Barros F., Vieyra A. (2001). Modulation of adenosine 5′-monophosphate adsorption onto aqueous resident pyrite: Potential mechanisms for prebiotic reactions. Orig. Life Evol. Biosph..

[44] Cody G.D., Boctor N.Z., Filley T.R., Hazen R.M., Scott J.H., Sharma A., Yoder H.S. (2000). Primordial carbonylated iron-sulfur compounds and the synthesis of pyruvate. Science.

[45] Cody G.D. (2004). Transition metal sulfides and the origins of metabolism. Annu. Rev. Earth Planet. Sci..

[46] Huber C., Wächtershäuser G. (1997). Activated acetic acid by carbon fixation on (Fe,Ni)S under primordial conditions. Science.

[47] Huber C., Wächtershäuser G. (1998). Peptides by activation of amino acids with CO on (Ni,Fe)S surfaces: Implications for the origin of life. Science.

[48] Russell M.J., Hall A.J., Gize A.P. (1990). Pyrite and the origin of life. Nature.

[49] Lill R. (2009). Function and biogenesis of iron–sulphur proteins. Nature.

[50] Eck R.V., Dayhoff M.O. (1966). Evolution of the structure of ferredoxin based on living relics of primitive amino acid sequences. Science.

[51] Meyer J. (2008). Iron-sulfur protein folds, iron-sulfur chemistry, and evolution. J. Biol. Inorg. Chem..

[52] McKibben M., Barnes H.L. (1986). Oxidation of pyrite in low temperature acidic solutions: Rate laws and surface textures. Geochim. Cosmochim. Acta.

[53] Team R.C. (2014). R: A Language and Environment for Statistical Computing.

[54] Guttenberg N., Virgo N., Chandru K., Scharf C., Mamajanov I. (2017). Bulk measurements of messy chemistries are needed for a theory of the origins of life. Philos. Trans. R. Soc. A Math. Phys. Eng. Sci..

[55] Van Veldhoven P.P., Mannaerts G.P. (1987). Inorganic and organic phosphate measurements in the nanomolar range. Anal. Biochem..

[56] Giri A., Choudhury M.D., Dutta T., Tarafdar S. (2012). Multifractal growth of crystalline NaCl aggregates in a gelatin medium. Cryst. Growth Des..

[57] Epstein I.R., Showalter K. (1996). Nonlinear chemical dynamics:  Oscillations, patterns, and chaos. J. Phys. Chem..

[58] Field R.J., Epstein I.R., Pojman J.A. (2000). An Introduction to Nonlinear Chemical Dynamics: Oscillations, Waves, Patterns, and Chaos.

[59] Wächtershäuser G. (2007). On the chemistry and evolution of the pioneer organism. Chem. Biodivers..

[60] Ruiz-Mirazo K., Briones C., de la Escosura A. (2014). Prebiotic systems chemistry: New perspectives for the origins of life. Chem. Rev..

[61] Semenov S.N., Kraft L.J., Ainla A., Zhao M., Baghbanzadeh M., Campbell V.E., Kang K., Fox J.M., Whitesides G.M. (2016). Autocatalytic, bistable, oscillatory networks of biologically relevant organic reactions. Nature.

