# Supplementary material for: Chemical Ecosystem Selection on Mineral Surfaces Reveals Long-Term Dynamics Consistent with the Spontaneous Emergence of Mutual Catalysis
Source: Life (Basel). 2019 Oct 23;9(4):80. doi: 10.3390/life9040080 (PMC6911371; doi:10.3390/life9040080)
Supplement: Supplementary file 1 [file life-09-00080-s001.zip › Life-590614_Supplemental_Data/Table_S1.pdf]

Table S1. Detailed composition of the final EPS soup.

Chemical Name	Vendor	CAS #	Concentration (mM)
**1,3-dihydroxyacetone**	Fisher Scientific	96-26-4	0.32
**2-aminobutyric acid**	Acros Organics	2835-81-6	0.08
**acetoguanamine**	VWR	541-02-9	0.16
**adenine**	Fisher Scientific	73-24-5	0.32
**adenosine triphosphate**	Sigma-Aldrich	34369-07-8	0.32
**ammonium chloride**	Sigma-Aldrich	12125-02-9	20
**ammonium persulfate**	IBI Scientific	7727-54-0	0.04
**β-alanine**	Tokyo Chemical	107-95-9	0.32
**butyric acid (sodium salt)**	Fisher Scientific	156-54-7	0.08
**cobalt(II) chloride anhydrous**	BTC Chemicals	7646-79-9	10^{-5}
**copper(II) chloride dihydrate**	Alfa Aesar	10125-13-0	10^{-5}
**cytosine**	Fisher Scientific	71-30-7	0.32
**D-(-)-ribose**	Fisher Scientific	50-69-1	0.16
**D-(+)-xylose**	Fisher Scientific	58-86-6	0.16
**D-(+)-glucose**	DOT Scientific Inc.	50-99-7	0.16
**DL-arabinose**	VWR	147-81-9	0.16
**formic acid 90%**	Aqua Solutions	64-18-6	0.64
**glycerol**	DOT Scientific Inc.	56-81-5	0.32
**glycolic acid**	Acros Organics	79-14-1	0.32
**hydroxybutyric acid**	Sigma-Aldrich	150-83-4	0.08
**iminodiacetic acid**	VWR	142-73-4	0.16
**L-alanine**	VWR	56-41-7	0.32
**L-arginine**	Sigma-Aldrich	74-79-3	0.16
**L-ascorbic acid**	DOT Scientific Inc.	50-81-7	0.04
**L-asparagine**	Acros Organics	5794-13-8	0.16
**L-aspartic acid**	Alfa Aesar	56-84-8	0.32
**L-cysteine**	DOT Scientific Inc.	52-90-4	0.16
**L-glutamic Acid**	VWR	56-86-0	0.32
**L-glutamine**	Sigma-Aldrich	56-85-9	0.16
**L-glycine**	Sigma-Aldrich	54-40-6	0.32
**L-histidine**	DOT Scientific Inc.	71-00-1	0.08
**L-isoleucine**	DOT Scientific Inc.	73-32-5	0.16
**L-leucine**	DOT Scientific Inc.	61-90-5	0.32
**L-lysine**	DOT Scientific Inc.	657-27-2	0.32
**L-methionine**	DOT Scientific Inc.	63-68-3	0.08
**L-phenylalanine**	DOT Scientific Inc.	63-91-2	0.16

**L-proline**	Alfa Aesar	147-85-3	0.16
**L-serine**	DOT Scientific Inc.	56-45-1	0.32
**L-threonine**	DOT Scientific Inc.	72-19-5	0.32
**L-tryptophan**	DOT Scientific Inc.	73-22-3	0.08
**L-tyrosine**	Amresco	60-18-4	0.16
**L-valine**	DOT Scientific Inc.	72-18-4	0.32
**lactic acid 88% solution**	Fisher Scientific	50-21-5	0.16
**magnesium chloride**	DOT Scientific In.	7791-18-6	50
**N-ethanolamine**	Sigma-Aldrich	141-43-5	0.08
**N-methylalanine**	Sigma-Aldrich	3913-67-5	0.08
**N-methylglycine**	Acros Organics	107-97-1	0.16
**N-methylurea**	Fisher Scientific	759-73-9	0.08
**nickel(II) chloride hexahydrate**	Chem-IMPEX Int'l	7791-20-0	4×10^{-4}
**nicotinamide**	DOT Scientific Inc.	98-92-0	0.08
**potassium chloride**	VWR	7447-40-7	10
**propionic acid**	Fisher Scientific	79-09-4	0.16
**pyruvic acid**	Fisher Scientific	127-17-3	0.08
**R-pantetheine**	Sigma-Aldrich	496-65-1	0.04
**sodium bisulfite**	Ward's	7631-90-5	0.08
**sodium chloride**	Fisher Scientific	7647-14-5	500
**sodium molybdate**	Strem Chemicals	10102-40-6	10^{-5}
**sodium nitrate**	Sigma-Aldrich	7631-99-4	20
**succinic acid**	Sigma-Aldrich	110-15-6	0.08
**succinonitrile**	VWR	110-61-2	0.16
**thymine**	Fisher Scientific	65-71-4	0.32
**uracil**	VWR	66-22-8	0.32
**urea**	IBI Scientific	57-13-6	0.16
**zinc(II) chloride**	Sigma-Aldrich	7646-85-7	1.5×10^{-5}
