# Supplementary material for: Chemical Ecosystem Selection on Mineral Surfaces Reveals Long-Term Dynamics Consistent with the Spontaneous Emergence of Mutual Catalysis
Source: Life (Basel). 2019 Oct 23;9(4):80. doi: 10.3390/life9040080 (PMC6911371; doi:10.3390/life9040080)
Supplement: Supplementary file 1 [file life-09-00080-s001.zip › Life-590614_Supplemental_Data/Table_S3.pdf]

Table S3. Mean absorbance values between 200 and 400 nm for the 10 experimental lineages and 10 controls at generations 6, 12, and 18 of experiment 1 (SE: standard error).

Generation 6

Wavelength	CTL Mean	EXP Mean	CTL SE	EXP SE
200	2.560433333	2.5698	0.006998393	0.004929526
205	2.7684	2.775466667	0.00727103	0.005115378
210	2.7364	2.7449	0.007613057	0.005700242
215	2.770633333	2.778366667	0.007281949	0.005623726
220	2.908866667	2.9131	0.007186889	0.005636476
225	3.0468	3.0502	0.008117815	0.004732369
230	3.510066667	3.525833333	0.012496986	0.013193015
235	2.313333333	2.3349	0.016131024	0.015919405
240	1.8835	1.900433333	0.015346586	0.013426734
245	1.9695	1.980633333	0.014857151	0.013930416
250	2.237333333	2.248233333	0.01449837	0.014822693
255	2.538833333	2.5507	0.013826312	0.015634142
260	2.7803	2.794333333	0.014289944	0.018052992
265	2.6189	2.635233333	0.013650713	0.016586884
270	2.253733333	2.2749	0.014143047	0.015621937
275	1.6432	1.665133333	0.014441505	0.013664934
280	1.082633333	1.102166667	0.015596538	0.012035167
285	0.673766667	0.693333333	0.015686103	0.010998567
290	0.4747	0.489266667	0.01524603	0.010430741
295	0.377133333	0.390333333	0.015190757	0.010131876
300	0.3279	0.340833333	0.015126573	0.009912863
305	0.283033333	0.2955	0.015033	0.009861297
310	0.255066667	0.267533333	0.014849257	0.009710436
315	0.234733333	0.247066667	0.014593563	0.009586164
320	0.2175	0.2301	0.014333172	0.009506486
325	0.202366667	0.2149	0.014054951	0.009378485
330	0.190833333	0.203366667	0.013780372	0.009282577
335	0.1817	0.194133333	0.013462146	0.009136431
340	0.1742	0.186766667	0.013209342	0.009059041
345	0.167033333	0.179466667	0.012901223	0.008924646
350	0.1608	0.1731	0.01260082	0.008742867
355	0.1543	0.1664	0.012283532	0.008573351
360	0.147833333	0.16	0.011931342	0.008376141
365	0.141366667	0.153433333	0.011542138	0.008228276

370	0.135	0.1468	0.011154515	0.007947773
375	0.128733333	0.1401	0.010697973	0.007687863
380	0.123566667	0.134866667	0.010296403	0.007480023
385	0.117666667	0.128566667	0.009910144	0.007143141
390	0.1135	0.124033333	0.009520244	0.006919187
395	0.1089	0.1191	0.009118903	0.006676386
400	0.105333333	0.115166667	0.008826019	0.00644995

Generation 12

Wavelength	CTL Mean	EXP Mean	CTL SE	EXP SE
200	2.544166667	2.515266667	0.007196773	0.00232209
205	2.760066667	2.7305	0.007770032	0.002651759
210	2.727833333	2.698766667	0.007984716	0.002741957
215	2.754433333	2.724366667	0.007738998	0.002241299
220	2.9166	2.8843	0.007730923	0.002235066
225	3.0858	3.052766667	0.00826705	0.003557352
230	3.179966667	3.141433333	0.007965028	0.007051471
235	2.058133333	2.013733333	0.009393639	0.006704962
240	1.7553	1.712	0.010323392	0.006382696
245	1.900833333	1.8604	0.011438805	0.007028006
250	2.160866667	2.123766667	0.012627163	0.007780675
255	2.439666667	2.405033333	0.013786054	0.008037939
260	2.6413	2.6091	0.015033759	0.008528894
265	2.462233333	2.4274	0.015412705	0.007587861
270	2.073633333	2.0365	0.015771606	0.006877785
275	1.474633333	1.4331	0.016016336	0.005765283
280	0.9562	0.910333333	0.016217293	0.005274263
285	0.5851	0.535933333	0.016253528	0.005192791
290	0.410266667	0.3598	0.016222188	0.00516055
295	0.324	0.272066667	0.015959503	0.005086188
300	0.278866667	0.225966667	0.015745268	0.005060101
305	0.241733333	0.188233333	0.01548206	0.004990344
310	0.218233333	0.164433333	0.015299889	0.004934374
315	0.200966667	0.147066667	0.015095469	0.004835128
320	0.186333333	0.1326	0.014909196	0.004803813
325	0.173066667	0.119966667	0.014632185	0.004731364
330	0.1634	0.110566667	0.014309044	0.00466376
335	0.1553	0.103166667	0.013978676	0.004532526
340	0.148866667	0.097166667	0.013686171	0.004444938

345	0.142833333	0.0916	0.013387306	0.004355749
350	0.137333333	0.087	0.012978021	0.004221851
355	0.1318	0.082333333	0.0125977	0.004125524
360	0.1256	0.077033333	0.012192996	0.00398079
365	0.120233333	0.072733333	0.011732161	0.003845099
370	0.114566667	0.068566667	0.011297097	0.003681396
375	0.109266667	0.0648	0.010875744	0.003505692
380	0.104766667	0.0617	0.010395085	0.003359472
385	0.1	0.058466667	0.009976342	0.003182175
390	0.095866667	0.055933333	0.00956887	0.002993911
395	0.091933333	0.0536	0.009208	0.002880788
400	0.088733333	0.051533333	0.008866849	0.002764138

Generation 18

Wavelength	CTL Mean	EXP Mean	CTL SE	EXP SE
200	2.573033333	2.501866667	0.025047299	0.008624302
205	2.787366667	2.7145	0.024773147	0.008562394
210	2.746933333	2.674	0.024930795	0.008935458
215	2.7726	2.697433333	0.025498875	0.009258138
220	2.924966667	2.8411	0.028385584	0.009645644
225	3.0548	2.955833333	0.032227743	0.012442779
230	3.127466667	2.979033333	0.03907769	0.017674713
235	1.9082	1.7473	0.046082628	0.01925158
240	1.486366667	1.3166	0.051957667	0.022540762
245	1.512366667	1.3262	0.057930622	0.026912364
250	1.666233333	1.462933333	0.063829327	0.031442825
255	1.881233333	1.6619	0.069269281	0.035559562
260	2.0393	1.810733333	0.072544722	0.037847611
265	1.9497	1.7315	0.067759874	0.034894367
270	1.692766667	1.4957	0.059579993	0.02879648
275	1.265966667	1.0942	0.050212203	0.021633893
280	0.9077	0.7583	0.044159197	0.016538623
285	0.619433333	0.483766667	0.041276416	0.013900936
290	0.4621	0.3328	0.041122153	0.01328677
295	0.373166667	0.2485	0.04187179	0.013230984
300	0.3248	0.203466667	0.042300447	0.013245479
305	0.287366667	0.168033333	0.042062553	0.013065981
310	0.263733333	0.146466667	0.041723779	0.012896391
315	0.2469	0.131	0.041348201	0.012817361

320	0.232566667	0.118333333	0.040947138	0.012683814
325	0.220466667	0.107533333	0.040547982	0.012530871
330	0.211266667	0.0998	0.040085017	0.012425261
335	0.204166667	0.094066667	0.039615107	0.012269407
340	0.198233333	0.089533333	0.03912049	0.012177337
345	0.192666667	0.085466667	0.038628087	0.012073191
350	0.187666667	0.082066667	0.038077834	0.01192761
355	0.182233333	0.078533333	0.037489284	0.011753786
360	0.1763	0.074666667	0.036832068	0.011558373
365	0.170666667	0.0713	0.036082339	0.011398727
370	0.164766667	0.067833333	0.035247421	0.011166803
375	0.1593	0.065033333	0.034367569	0.010914071
380	0.154066667	0.0626	0.033433918	0.010631527
385	0.149	0.0603	0.03253345	0.010334567
390	0.1443	0.058233333	0.03157633	0.010065345
395	0.139966667	0.056533333	0.030737772	0.00979784
400	0.135666667	0.0547	0.029892535	0.009534615
