# Supplementary material for: Chemical Ecosystem Selection on Mineral Surfaces Reveals Long-Term Dynamics Consistent with the Spontaneous Emergence of Mutual Catalysis
Source: Life (Basel). 2019 Oct 23;9(4):80. doi: 10.3390/life9040080 (PMC6911371; doi:10.3390/life9040080)
Supplement: Supplementary file 1 [file life-09-00080-s001.zip › Life-590614_Supplemental_Data/Table_S2.pdf]

Table S2. Outputs of ANOVA and Tukey-HSD tests used to determine the statistical significance of differences among controls in experiment 2.

**One-Way ANOVA**	Df	Sum Sq	Mean Sq	F-value	*p*-value
Generation	3	4569	1523	8.843	<0.001
Residuals	35	6028	172.2		

**Tukey-HSD Post-Hoc Test**	*p*-value
gen18-gen12	0.24
gen6-gen12	0.05
gen6-gen18	0.85
gen6-gen24	0.09
gen24-gen12	0.00
gen24-gen18	0.01
