# Supplementary material for: Chemical Ecosystem Selection on Mineral Surfaces Reveals Long-Term Dynamics Consistent with the Spontaneous Emergence of Mutual Catalysis
Source: Life (Basel). 2019 Oct 23;9(4):80. doi: 10.3390/life9040080 (PMC6911371; doi:10.3390/life9040080)
Supplement: Supplementary file 1 [file life-09-00080-s001.zip › Life-590614_Supplemental_Data/Figure_S3.pdf]

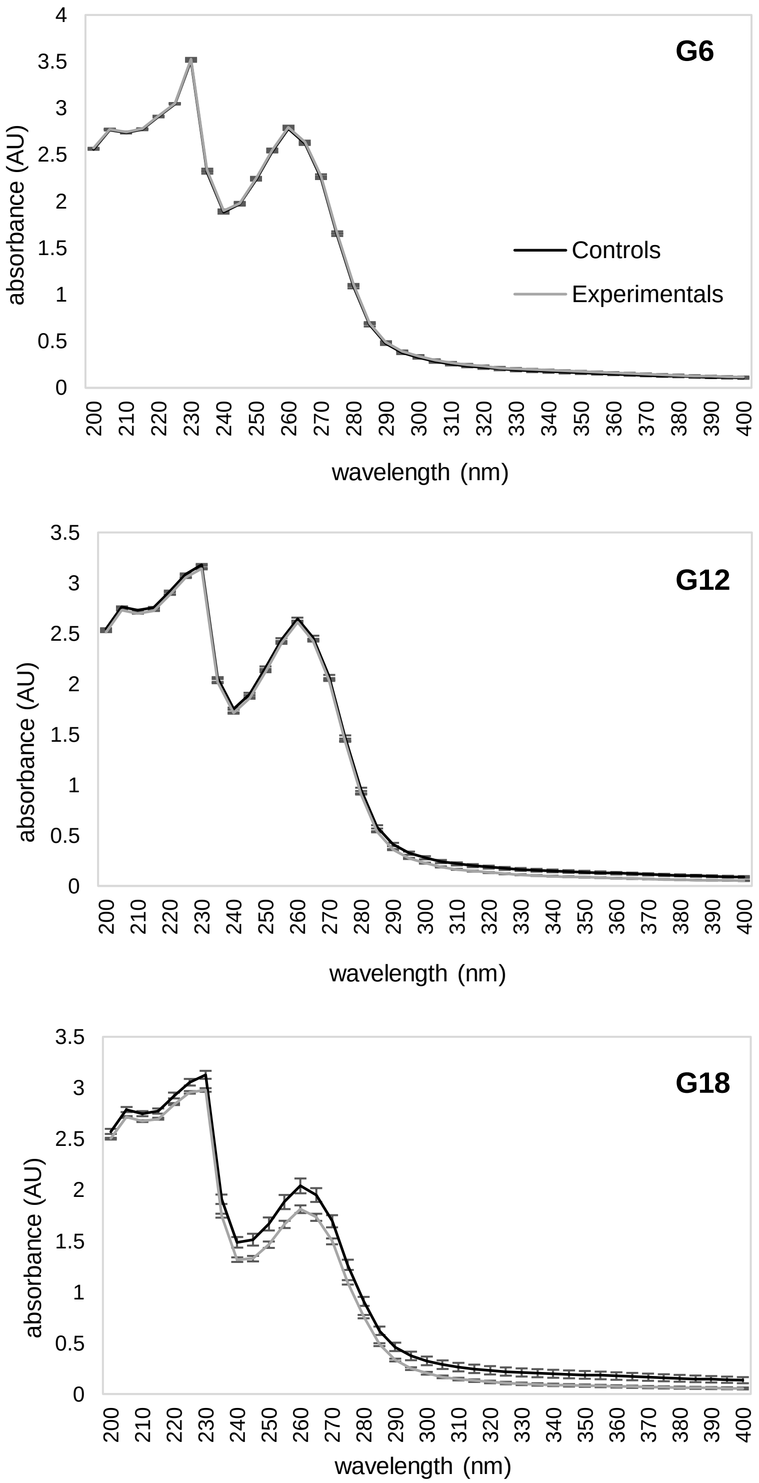

Figure S3: Average UV-Vis absorbance spectra of control (black line) and experimental (grey line) lineages at generations 6, 12, and 18. Error bars represent the standard error of the mean.
