# Supplementary material for: Chemical Ecosystem Selection on Mineral Surfaces Reveals Long-Term Dynamics Consistent with the Spontaneous Emergence of Mutual Catalysis
Source: Life (Basel). 2019 Oct 23;9(4):80. doi: 10.3390/life9040080 (PMC6911371; doi:10.3390/life9040080)
Supplement: Supplementary file 1 [file life-09-00080-s001.zip › Life-590614_Supplemental_Data/Figure S2.pdf]

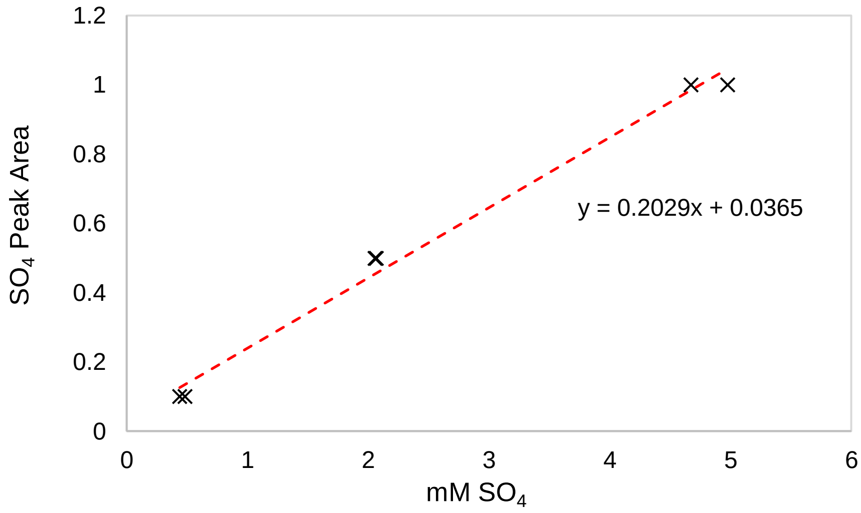

Figure S2: Ion chromatography sulfate standard curve used to determine the amount of sultate released by pyrite mineral to estimate the extent of pyrite oxidation after acid washing. Pyrite washed using the protocol described in the main text produced >0.05 mM SO_4.
