# Supplementary material for: Chemical Ecosystem Selection on Mineral Surfaces Reveals Long-Term Dynamics Consistent with the Spontaneous Emergence of Mutual Catalysis
Source: Life (Basel). 2019 Oct 23;9(4):80. doi: 10.3390/life9040080 (PMC6911371; doi:10.3390/life9040080)
Supplement: Supplementary file 1 [file life-09-00080-s001.zip › Life-590614_Supplemental_Data/Figure_S1.pdf]

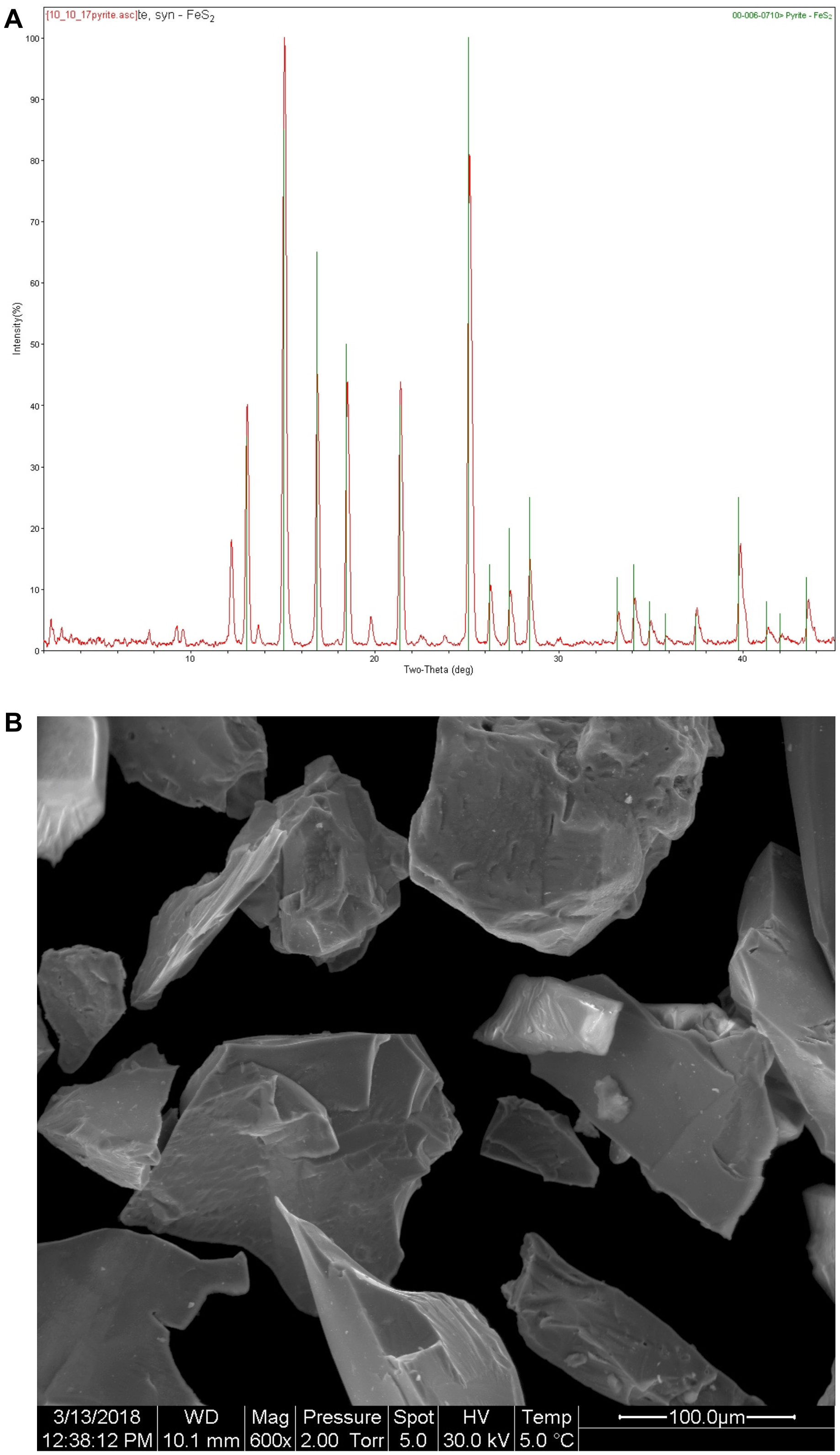

Figure S1: (A) X-ray diffractogram of washed high-grade natural pyrite powder (50 kV; 50 mA; 60 min exposure). (B) ESEM of washed pyrite grains (30 kV; 5.0 spot; 2.00 torr).
