# Supplementary material for: Chemical Ecosystem Selection on Mineral Surfaces Reveals Long-Term Dynamics Consistent with the Spontaneous Emergence of Mutual Catalysis
Source: Life (Basel). 2019 Oct 23;9(4):80. doi: 10.3390/life9040080 (PMC6911371; doi:10.3390/life9040080)
Supplement: Supplementary file 1 [file life-09-00080-s001.zip › Life-590614_Supplemental_Data/Figure_S5.pdf]

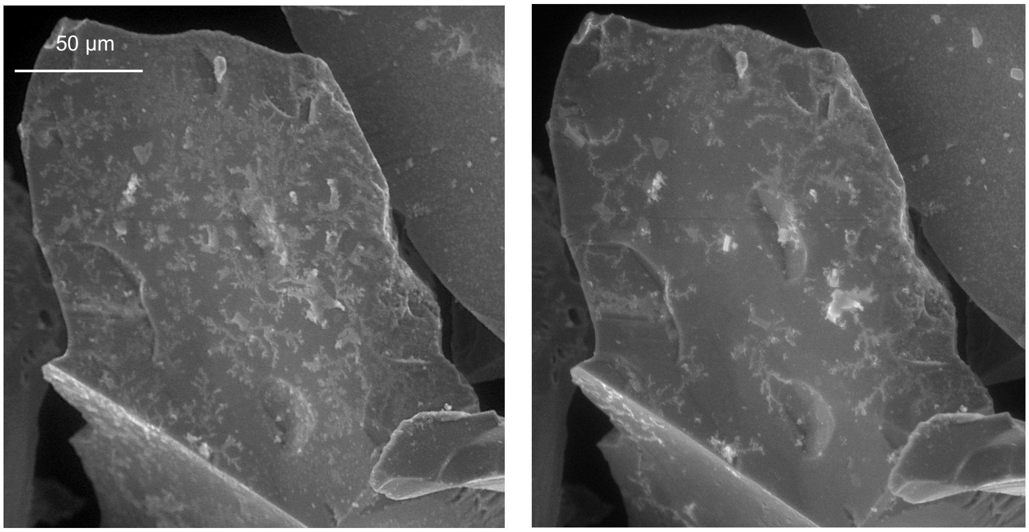

A　　　　　　　　B

Figure S5. Comparison of fractals (A) before and (B) after raising and re-lowering the water vapor pressure in the ESEM chamber. Raising the vapor pressure above 6.5 torr lead to the dissolution and disappearance of these structures (not shown). Note that fractals are present in (B), although to a lesser extent, which indicates that they dissolved and then re-precipitated during drying.
