# Supplementary material for: Chemical Ecosystem Selection on Mineral Surfaces Reveals Long-Term Dynamics Consistent with the Spontaneous Emergence of Mutual Catalysis
Source: Life (Basel). 2019 Oct 23;9(4):80. doi: 10.3390/life9040080 (PMC6911371; doi:10.3390/life9040080)
Supplement: Supplementary file 1 [file life-09-00080-s001.zip › Life-590614_Supplemental_Data/Figure_S4.pdf]

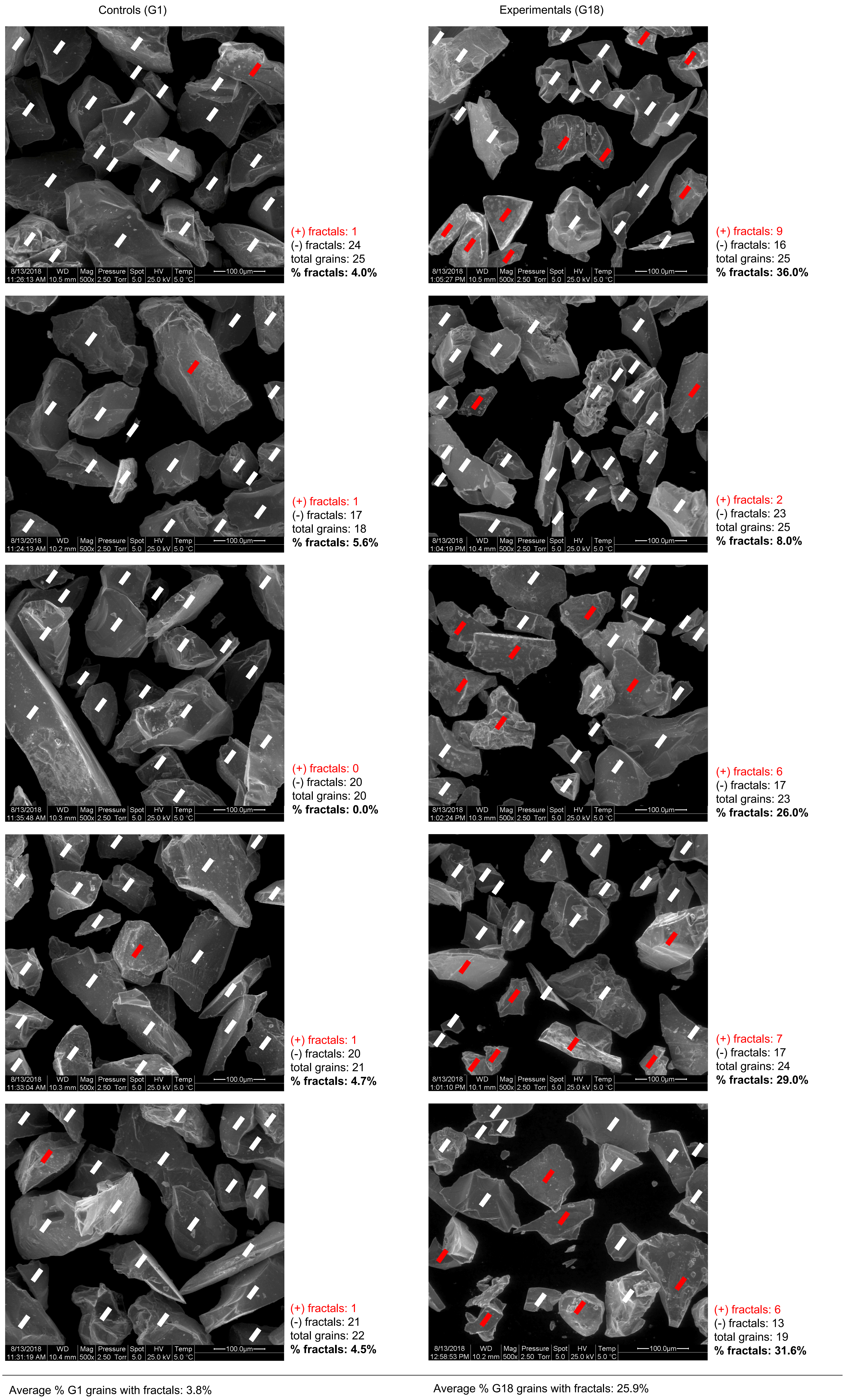

Figure S4. Example of the image scoring strategy used to estimate the percentage of grains with fractal structures on one experimental and one control replicate imaged by SEM. Grains with red marks have fractals; grains with white marks have no visible fractals. The percentage of grains with fractals is calculated in 5 random fields at 500X and averaged to estimate the total percentage of grains with fractals for each replicate.
